# Improving UK data on avoidable perinatal brain injury: review of data dictionaries and consultation

**DOI:** 10.1038/s41390-025-03842-3

**Published:** 2025-01-30

**Authors:** Jan W. van der Scheer, Victoria Komolafe, Kirstin Webster, Stamatina Iliodromiti, Charles C. Roehr, Asma Khalil, Tim Draycott, Louise Dewick, George Dunn, Rachel Walsh, Philip Steer, Alessandra Giusti, Mark L. Cabling, Nick Fahy, ABC Contributor Group, Alissa E. Frémeaux, Amar M. Karia, Annette Anderson, Bertie Leigh, Chris Gale, Cora Doherty, Daniel Wolstenholme, James Walker, Julia Gudgeon, Laura Cowell, Marian Knight, Matthew C. Jolly, Muhammed Ally Hussein Wahedally, Tim J. van Hasselt, Tina Harris, Mary Dixon-Woods

**Affiliations:** 1 THIS Institute (The Healthcare Improvement Studies Institute), University of Cambridge, Strangeways Research Laboratory, Cambridge, CB1 8RN UK; 2 Royal College of Midwives, 10-18 Union Street, London, SE1 1SZ UK; 3 Royal College of Obstetricians & Gynaecologists, 10-18 Union Street, London, SE1 1SZ UK; 4 Department of Population Sciences, University of Leicester, Leicester, LE1 7RH UK; 5 Women’s Health Research Unit, Wolfson Institute of Population Health, Queen Mary University London, London, UK; 6 University of Bristol, Faculty of Health Sciences, Bristol, UK; 7 National Perinatal Epidemiology Unit, Clinical Trials Unit, Oxford Population Health, Medical Sciences Division, University of Oxford, Oxford, UK; 8 North Bristol NHS Trust, Bristol, BS10 5NB UK; 9 NHS Resolution, E14 4PU London, UK; 10 RAND Europe, Westbrook Centre Milton Road, Cambridge, CB4 1YG UK; 11 Hempsons, London, UK; 12 Imperial College London, London, UK; 13 Health Services Safety Investigations Body, Dorset, UK; 14 NHS England, London, UK; 15 University of Oxford, Oxford, UK; 16 University of Leicester, Leicester, UK; 17 De Montfort University, Leicester, UK

## Abstract

**Background:**

High quality data is important to understanding epidemiology and supporting improvement efforts in perinatal brain injury. It is not clear which data items relevant to brain injury are captured across UK sources of routinely collected data, nor what needs to be done to ensure that those sources are fit for purpose in improving care.

**Methods:**

We reviewed data dictionaries of four main UK perinatal data sources and consulted a multi-professional group (*N* = 27) with expertise in neonatal/maternity care, statistics, and clinical negligence.

**Results:**

None of the data sources we reviewed currently captures, on its own, the range of items relevant to brain injury. Data items lack common definitions and ongoing linkage across the different sources. Our consultation identified the need for standardising the definition of avoidable perinatal brain injury, resolving inconsistencies in capturing data, improving linkage of data across existing data sources, and co-designing a strategy for meaningful use of data.

**Conclusions:**

Limited standardisation and linkage across UK data sources are key problems in using data to guide improvement efforts aimed at reducing risk of avoidable perinatal brain injury. A programme involving co-design with healthcare professionals and families to improve capture and use of data is now needed.

**Impact:**

- Limited standardisation and linkage across UK data sources currently challenge the use of data as the basis of efforts to reduce risk of avoidable perinatal brain injury.
- A harmonisation programme involving consultation and co-design with healthcare professionals, families, and other specialists is needed to enable better capture and use of data in this key area.
- There is need to standardise the definition of avoidable perinatal brain injury, resolve inconsistencies in capturing data, improve linkage of data collected across existing data sources, and co-design a strategy for meaningful use of data.

## Introduction

Potentially avoidable brain injury during perinatal care can cause devastating consequences for babies and their families,^1–3^ along with significant lifetime costs for health and care services.^4^ In the UK, successful clinical negligence claims for perinatal brain injury can result in settlement costs exceeding £20 million per avoidable case of cerebral palsy in the National Health Service (NHS).^5,6^ Perinatal brain injury concerns brain damage occurring during or soon after birth, including conditions such as intraventricular haemorrhage, perinatal stroke, and hypoxic-ischaemic encephalopathy (HIE).^7^ While its definition continues to be debated,^8,9^ HIE commonly refers to a brain injury caused by a series of fetal and neonatal insults around the time of birth^10^ that may be avoidable under conditions of optimal antenatal and intrapartum care.^5,6,11–13^ With estimates of average incidence ranging from 1.5 to 2.0 per 1000 live births,^7,14,15^ HIE is one of the leading causes of neurodisability and mortality in near-term and term babies.^1,2^

High quality data are needed for effective clinical audit and service improvement,^15–24^ and to enable understanding of the epidemiology related to incidence and risk factors for avoidable perinatal brain injury associated to HIE.^7,15,19–21,24,25^ Such data are especially important in identifying unwarranted variation across settings and over time, establishing targets for improvement, learning from positive deviance, building feedback loops with healthcare units about their performance, and informing the design, development and testing of improvement efforts.^16–18,21–23,25–27^ While data on perinatal care are routinely collected in the UK,^19,20,28,29^ challenges remain in relation to reliability and integration,^20,30,31^ inclusion and presentation of data most relevant to families,^26,32,33^ and deployment in data-driven improvement efforts.^21^ These challenges are similar to those in other national and international efforts to improve reporting and use of routinely collected data on perinatal injury,^22,24,34–39^ including HIE.^10,25^

A range of national data sources (i.e. datasets and audits) captures data relevant to perinatal brain injury associated to HIE in the UK.^19,20,28–31,40–47^ However, it remains unclear which data items relevant to incidence or risk factors are currently available across the data sources, and whether the data definitions used across the various data dictionaries are comparable.^48^ It is also not clear how challenges in collecting, recording and reporting of data might best be addressed. Accordingly, we aimed to:

1. identify and compare data items and their definitions relevant to monitoring incidence or risk factors in data dictionaries of perinatal data sources in the UK,
2. elicit multi-professional views on data items and an avoidable brain injury definition most relevant for an integrated UK data source, and
3. explore multi-professional views on challenges and optimisation of capturing and using routinely collected data in the UK.

## Methods

As part of a quality improvement effort (see Acknowledgements), we conducted a review of relevant data dictionaries and consulted a multi-professional group.

### Review of data dictionaries

National data sources were selected based on their relevance and size, including the Maternity Services Data Set (MSDS),^28,31,40^ the Hospital Episode Statistics for Admitted Patient Care (HES APC),^29,41,42^ the National Neonatal Research Database (NNRD),^19,43,44^ and the National Maternity and Perinatal Audit (NMPA)^20,30,45^ (Table 1). The data dictionary of the National Neonatal Audit Programme (NNAP)^46,47^ was not separately reviewed as it uses data extracted from the same source as the NNRD (Table 1).

**Table 1:** Main data sources (i.e. dataset and audits) used in UK perinatal care settings in the National Health Service (NHS) that include routinely collected data relevant to clinical indicators or risk factors for avoidable perinatal brain injury associated to HIE.

Data source	Organisation	Type of data source	Data	Time period
Maternity Services Data Set (MSDS)^28,31,40^	Commissioned by a national governing body (NHS England)	Single data source used in maternity care. Includes patient-level data about maternity services activities, funded and/or provided by the NHS in England, relating to mothers and babies, from the first antenatal appointment (booking) through to discharge from maternity services.	Data items and definitions are extracted from local electronic patient record platforms used in maternity units, and sent to NHS England for analysis.	2015–2019: MSDS version 1.5 (version 1.0 did not come into operation). 2019 to current: MSDS version 2.0, including mandated submission of all relevant maternity records.
Hospital Episode Statistics for Admitted Patient Care (HES APC)^29,41,42^	Commissioned by a national governing body (NHS England)	Single data source used in hospital admissions. Described as a “data warehouse” with records of information on all NHS hospital admissions in England.	Data on “birth episodes”, i.e. any record that contains valid information about the mode of birth in either the HES “maternity tail” or the procedure fields. Data include patient demographics, admission and discharge dates, and diagnostic and procedure codes.	Established in 1989, currently in operation.
National Neonatal Research Database (NNRD)^19,43,44^	Founded and managed by the Neonatal Medicine Research Group at Imperial College London	Single data source used in neonatal care. Holds clinical data routinely captured during all admissions to NHS neonatal units in England, Scotland, Wales and the Isle of Man.	Data are extracted from neonatal units using their electronic patient record platform. Only babies who are admitted to neonatal care will have an NNRD record.	Established in 2007, currently in operation.
National Maternity and Perinatal Audit (NMPA) from 2015 to 2017^20,30,45^	Commissioned by a national clinical audit governing body (Healthcare Quality Improvement Partnership), and led by the Royal College of Obstetricians and Gynaecologists in partnership with the Royal College of Midwives, the Royal College of Paediatrics and Child Health and the London School of Hygiene and Tropical Medicine	Linked data source used for audit of NHS maternity services across England, Scotland and Wales, reporting on a suite of processes and outcomes of maternity and perinatal care.	Data include specific extracts of other data sources, i.e. electronic records from local maternity units (given that MSDS data was not yet available), linked with HES APC and the NNRD	1 April 2015 to 21 March 2017: extracts of NNRD data linked with extracts of local electronic patient record platforms used in maternity units
National Maternity and Perinatal Audit (NMPA) after 2017^20,30,45^	As above for NMPA 2015–2017	As above for NMPA 2015–2017	Data include specific extracts of other data sources, i.e. MSDS and HES APC (linkage to NNRD not feasible)	1 April 2017 to 31 March 2019: extracts of MSDS version 1.5 linked with HES APC. 1 April 2019 to current: extracts expected to be used from MSDS version 2.0 and linked with HES APC
National Neonatal Audit Programme (NNAP)^20,47^	Commissioned by a national clinical audit governing body (Healthcare Quality Improvement Partnership), and delivered by the Royal College of Paediatrics and Child Health	Single data source used in neonatal care. Holds clinical data required for the audit’s reporting that are routinely captured during all admissions to NHS neonatal units in England, Scotland, Wales and the Isle of Man.	Data are extracted from neonatal units using their electronic patient record platform. Only babies who are admitted to neonatal care will be included in NNAP outputs.	Established in 2006, currently in operation.

All databases utilise coding from the OPCS Classification of Interventions and Procedures (OPCS-4), Systematised Nomenclature of Medicine - Clinical Terms (SNOMED CT) and International Classification of Diseases (ICD-10) coding systems.

The origins of the reviewed data sources are variable (Table 1), including “warehouses” of routinely collected data managed by NHS England. The MSDS, for example, re-uses clinical and operational data from the time of booking appointment to discharge from maternity care, while the HES APC includes data on episodes of care for patients admitted to hospital. By contrast, the NNRD, which captures specific data items about the care of all babies admitted to NHS neonatal care across England, Scotland, Wales and the Isle of Man, is managed by a research group based in a university. Finally, the NMPA, led by a clinical consortium including the Royal College of Obstetricians and Gynaecologists, is a large-scale national clinical audit that assesses specific care processes and outcomes using linked maternity data from the MSDS, HES APC and/or NNRD.

We reviewed the data dictionaries of the MSDS, HES APC, NNRD and NMPA to:

(i) identify which of their data items are relevant to clinical indicators or risk factors of avoidably perinatal brain injury associated to HIE, and
(ii) assess homogeneity of definitions of similar data items across the data sources.

We manually searched the data sources’ dictionaries and associated International Classification of Diseases 10^th^ revision (ICD-10) codes. We mapped the identified data items to a longlist of potentially pertinent clinical indicators and risk factors, which we generated based on authorial expertise, national guidelines, and literature review.^6,7,24,49–60^ This longlist included potential clinical indicators of injury (e.g. Apgar scores, resuscitation, therapeutic hypothermia), antenatal risk factors (e.g. tobacco smoking, suspected fetal growth restriction, pre-eclampsia), and intrapartum risk factors (e.g. gestational age, maternal pyrexia, mode of birth).^6,7,49–61^ Findings were summarised in colour-coded tables with clinical indicators (Table 2) or antenatal/intrapartum risk factors (Table 3).

**Table 2:** Clinical indicators of avoidable brain injury associated to hypoxic ischaemic encephalopathy (HIE): potentially relevant data items that were identified in one or more of the data sources.

	MSDS version 2.0	HES APC	NNRD	NMPA 2015–2017	NMPA 2017–2019
Baby alive or dead at birth	Y	Y	N	Y	Y
Apgar score at one and five minutes	Y	N	Y	Y	Y
Time to first spontaneous breathing	N	N	Y	Y	N
Resuscitation	Y	Y	Y	Y	Y
Resuscitation method (bag and mask, intubation)	Y	Y	Y	Y	Y
Umbilical cord blood pH, arterial, venous, base deficit/lactate	N	N	Y	Y	N
Seizures	Y	Y^a^	Y	Y	Y
Transfer to neonatal unit	Y	Y	Y	Y	Y
Therapeutic hypothermia	N	N	Y	Y	N
Neurology – Central tone	N	Y^a^	Y	Y	Y^a^
Neurology – Consciousness	N	Y^a^	Y	Y	Y^a^
Magnetic resonance imaging (MRI) scan	N	N	Y	Y	N
Cerebral function monitoring (CFM) or electroencephalogram (EEG)	N	N	Y	Y	N
Hypoxic-ischaemic encephalopathy (HIE)	N	Y^a^	Y	Y	Y^a^

*HIE* hypoxic ischaemic encephalopathy, *MSDS* Maternity Services Data Set (MSDS),^28,31,40^ *HES APC* Hospital Episode Statistics for Admitted Patient Care,^29,41,42^ *NNRD* National Neonatal Research Database,^19,43,44^ *NMPA* National Maternity and Perinatal Audit.^20,30,45^

^a^Derived from ICD-10 code. ^#^Available via ICD-10 code if sepsis confirmed.

**Table 3:** Risk factors of avoidable brain injury associated to HIE: potentially relevant data items that were identified in one or more of the data sources.

Maternal characteristics	MSDS version 2.0	HES APC	NNRD	NMPA 2015–2017	NMPA 2017–2019
Ethnic group	Y	Y	Y	Y	Y
Parity	Y	Y	Y	Y	Y
Maternal age	Y	Y	Y	Y	Y
Tobacco smoking	Y	N	Y	Y	Y
Indication of social status (e.g. Carstairs, single unsupported mother, Index of Multiple Deprivation)	Y	Y	Y	Y	Y
**Antenatal risk factors**					
Height, weight and BMI at first antenatal appointment (booking)	Y	N	N	Y	Y
Previous caesarean birth	Y	Y	N	Y	Y
Previous perinatal loss (stillbirth/neonatal death)	Y	Y	N	Y	Y
Previous SGA baby <10^th^ centile	N	Y	N	Y	Y
Gestation at booking	Y	Y	N	Y	Y
Maternal diabetes (gestational and pre-pregnancy)	Y	Y^a^	Y	Y	Y
Cardiac disease	Y	Y^a^	Y	Y	Y
Epilepsy	Y	Y^a^	Y	Y	Y
Group B Streptococcus (GBS) carrier	Y	N	Y	Y	Y
Suspected fetal growth restriction	N	N	Y	Y	N
Pre-eclampsia (blood pressure ≥140/90 mmHg, proteinuria ≥2+, pre-existing or pregnancy induced hypertension)	Y	Y^a^	Y	Y	Y
Antepartum bleeding	Y	Y^a^	Y	Y^a^	Y
**Intrapartum risk factors**					
Gestational age (weeks + days)	Y	Y	Y	Y	Y
Mode of birth: spontaneous, instrumental, vaginal breech, planned caesarean, emergency caesarean	Y	Y	Y	Y	Y
Labour onset – spontaneous, no labour, or induced (prostaglandins or balloon or artificial rupture of membranes or oxytocin)	Y	Y	Y	Y	Y
Presentation at onset of labour or birth (e.g. cephalic, breech)	Y	N	Y	Y	Y
Chorioamnionitis	N	Y^a^	Y	Y^a^	Y^a^
Augmentation with oxytocin	Y	N	Y	Y	Y
Duration of rupture of membranes	Y	N	Y	Y	Y
Duration of first stage of labour	Y	N	N	Y	Y
Duration of second stage of labour	Y	N	N	Y	Y
Analgesia, including anaesthesia in labour	Y	Y	Y	Y	Y
Antibiotics given in labour	N	N	Y	Y	Y
Maternal pyrexia	N	N^b^	Y	Y	N^b^
Meconium staining of the amniotic fluid	N	N	Y	Y	N
Intrapartum bleeding	Y	Y^a^	N	Y^a^	Y

*HIE* hypoxic ischaemic encephalopathy, *MSDS* Maternity Services Data Set (MSDS),^28,31,40^ *HES APC* Hospital Episode Statistics for Admitted Patient Care,^29,41,42^ *NNRD* National Neonatal Research Database,^19,43,44^ *NMPA* National Maternity and Perinatal Audit.^20,30,45^

^a^Derived from ICD-10 code.

^b^Available via ICD-10 code if sepsis confirmed.

### Multi-professional consultation

We conducted a consultation with a multi-professional group with expertise in a range of relevant fields, including neonatal care, maternity care, statistics, and clinical negligence. Using individual interviews, the goal was to explore views on relevant data items and definitions of avoidable injury as well as current challenges in capturing data (i.e. collecting, recording and reporting data), including:

- definitions of avoidable perinatal brain injury
- data items indicative of presence of avoidable injury
- data items on antenatal and intrapartum risk factors relevant to avoidable injury,
- feasibility of reliable data collection across healthcare units, electronic patient record platforms and data sources, and
- use of data to support improvement in care.

We employed a purposive sampling technique, with the aim of achieving a diversity of professional perspectives.^62^ Discussion among the authorial team (consisting of a range of experts on perinatal brain injury and data collection) had identified neonatologists, obstetricians, midwives, solicitors, statisticians, and academics as having expertise most relevant for the topic of the consultation. Suitable participants were identified using the authorial team’s professional networks and knowledge of the field. The principle of information power was used to confirm that the consultation captured a sufficient range and depth of views.^63^

Trained interviewers with a clinical background in neonatal or maternity care (VK, KW, MS, LD, RW) used a prompt guide (Supplementary Material 1), which had been developed through group discussion and pilot testing among the authorial team. The interviews (all conducted online and lasting 22–90 min) were transcribed verbatim and anonymised. Analysis used a thematic framework based on the prompt guide and on cross-cutting themes generated through inspection of the transcripts (see details in Supplementary Material 2).

Review by an institutional review board/ethics committee was not required, as the consultation was an engagement exercise classified as a quality improvement activity,^64^ in which all participants were invited to join the authorial team or a contributor group if they wished to be named. All participants provided written informed consent, including permission to publish anonymised quotes and synthesis of their expressed views.

## Results

### Review of data dictionaries

Tables 2 and 3 show that none of the four reviewed data sources on its own captures the range of items potentially relevant to avoidable perinatal brain injury. For example, the two sources that capture data from maternity services (MSDS and HES APC) do not systematically include clinical indicators for brain injury (Table 2), since maternity settings are not typically where the diagnosis is made. In contrast, the NNRD does hold a range of clinical indicators of brain injury from all babies admitted to UK neonatal care (Table 2), but is limited in capturing antenatal and intrapartum data items (Table 3). Moreover, the NNRD does not capture data on babies with mild HIE (a condition that may result in impaired brain development in up to one in five babies)^65^ who are usually managed on postnatal or paediatric wards rather than being admitted to neonatal care. This likely results in under-reporting of mild HIE and associated risk factors.

Ongoing linkage between the data sources to enable continuous monitoring of integrated data is lacking. The NMPA has used local maternity records, HES APC data, and NNRD data to link a range of relevant data from mothers and babies across set periods (e.g. in 2015–2017 using local records and NNRD data and later in 2017–2019 using MSDS and HES APC, see Table 1). However, this practice is not routinised to ensure ongoing linkage of all available data of maternal and neonatal data. Further complicating the picture, operational definitions of similar data items differ across the data sources.

Further, most intrapartum risk factors for perinatal brain injury identified by National Institute for Health and Care Excellence (NICE)^54^ – including fetal heart rate features, delay in labour, meconium-stained liquor, vaginal bleeding, maternal pyrexia, and tachysystole^49–54^ – are not consistently captured within any of the data sources we reviewed (Table 3).

### Multi-professional consultation

Twenty-seven participants took part in the interview consultation (Table 4). Analysis of their views generated seven themes relating to current challenges in using data to guide improvement efforts aimed at reducing risk of perinatal brain injury (Table 5).

**Table 4:** Professional background of the multi-professional group (*N* = 27) taking part in the consultation.

Professional background	*n*
**Neonatology**	
Consultant neonatologists with senior academic/policy position	4
Neonatal clinical fellows with academic/policy position	2
Registrar neonatologist with an academic/policy position	1
**Maternity care**	
Consultant obstetrician with senior academic or national policy positions	6
Midwife with senior academic or national policy position	5
Senior digital midwife	3
Registrar obstetrician with Royal College position	2
**Other**	
Solicitor specialising in clinical negligence	2
Statistician working with national maternity and neonatal data	1
Senior academic in maternal/child public health	1

**Table 5:** Multi-professional views (*N* = 27): synthesis relating to addressing current challenges to using data to guide improvement efforts aimed at reducing risk of perinatal brain injury.

Theme	Synthesis of multi-professional views
1. Standardised definition of potentially avoidable perinatal brain injury	• Lack of a standardised, consensus-built definition of potentially avoidable perinatal brain injury limits the ability to effectively monitor incidence and risk factors. • Frequently suggested definitions focused on hypoxic-ischaemic encephalopathy, which was seen as a potentially avoidable condition in ideal care conditions (Table 6). • Related or other frequently suggested definitions referred to causes such as absent, poor, late or suboptimal clinical management during perinatal, antenatal or intrapartum care (Table 6). • Questions relating to avoidability should consider whether the outcome would have been different in a different healthcare unit, with different healthcare professionals, or using different clinical practices, while remaining cognisant of the challenges in determining avoidability.
2. Agreed set of data items relevant for monitoring incidence and risk factors	• An agreed set of data items relevant for monitoring incidence and risk factors of potentially avoidable perinatal brain injury, including postpartum follow-up, is needed to generate an integrated national data source. • A large range of risk factors and diagnostic features for potential use in an integrated data source can be identified (Table 7). • Follow-up of babies with potential brain injury is vital, since injury or developmental challenges may present some time after birth, even if there is no early indication of injury. • Long-term follow-up may be challenging, but might be mitigated through use of existing community services (e.g. health visitors) and parents’ reports.
3. Addressing inconsistency and subjectivity across data sources and healthcare units	• Duplicative recording of data items across multiple data sources is common but should be avoided. • To enable and optimise an integrated data source, the inconsistency and subjectivity involved in capturing many data items relevant to brain injury (e.g. therapeutic hypothermia criteria, Apgar scores, fetal growth restriction, intrapartum risk factors) should be addressed. • Inconsistency may be associated to use of different types of input fields in the various electronic patient record platforms used across healthcare units, and varying data definitions of similar data items across data sources. • Subjectivity arises partly because units and professionals differ in cardiotocography (CTG) classifications, have difficulties in defining a standardised parameter for risk factors that can evolve during labour, and differently interpretate results of some diagnostic procedures, such as brain magnetic resonance imaging (MRI) and continuous electroencephalography (EEG) monitoring. • Resolving some of the subjectivity would likely require more evidence on the robustness of clinical indicators and risk factors associated to perinatal brain injury.
4. Systematic linkage and integration of data items across data sources and platforms	• A programme of work is needed to operationalise and standardise operational definitions that are currently not harmonised across data sources and associated electronic patient record (EPR) platforms. • Ways to improve linkage of the data sources may include incentivising EPR platform suppliers to facilitate data linkage, “nudging” more communication between neonatal and maternity teams who have access to different data sources, and linking the baby’s NHS number to the mother’s health record. • Future steps for integration could consider linking perinatal data to post-perinatal data that are usually captured in separate paediatric data sources.
5. Organisational change to improve consistent, reliable and feasible data collection	• Systems change, socio-technical change and culture change are needed to enable consistent and reliable data collection that is useful for improving care. • Change should start with an assessment on needs and required resources for training, data entry, data management, data analysis, and quality assurance. • Change may be enabled by prioritising recording of clinical data over administrative data, e.g. about costs. • Making the data collection system more user-friendly, for example by focusing on default settings, mandatory inputs and electronic (non-human) interpretations and enabling some pragmatism, may support improvement. • Solutions may be found in making relevant data items mandatory in perinatal data collections, a coding framework for text-based data, and use of innovative technology for standardised interpretation of data instead of relying on subjective interpretation. • Data should mostly be recorded contemporaneously (in real time as part of routine care) rather than retrospectively, to reduce risks of poor data quality and professional burden – this could be achieved in various ways (Supplement 3).
6. Engaging, training and funding healthcare professionals involved in data capture	• Engaging with all relevant healthcare professionals is needed to reach shared understanding about the rationale and importance of the data. • Effective communication is needed to mitigate the risk that professionals may think that data could be used “against them” e.g. in the event of controversy. • “Professionalising” data capture could be supported by training so that it becomes part of clinicians’ skillsets. • Funding and resources are needed for data management and should accommodate adequate time, training, and resources for digital midwives, nurses, neonatologists, paediatricians, informatics teams, data quality managers, and others dedicated to lead or support reliable data capturing. • Funding allocation should be subject to the number and complexity of data items collected.
7. Co-designing systems with healthcare professionals and families to improve use of data	• Co-design with healthcare professionals and families is needed to ensure data presentation and use is relevant, acceptable and becomes part of everyday practice, not an “extra thing”. • Comparing unit data to national data generates possible risks of blame dynamics, since national benchmarking may not sufficiently consider local populations and challenges unique to each unit. • Sensible comparative methods need to be co-designed with healthcare professionals and families – for example co-design of feedback loops that combine individual case reviews with “big data” comparisons and priorities of families. • Data exchange from local to national entities should utilise existing auditors to support local-to-national data exchange, and not cause further strain on healthcare services and professionals. Some national oversight of feedback loops might be helpful, but data feedback should primarily be a local enterprise. • Where possible, data should be accessible to anyone, not just to those at senior levels.

The participants viewed the current lack of a standardised, consensus-built definition of avoidable perinatal brain injury as an important problem in effectively monitoring incidence and risk factors (Table 5 – theme 1). Frequently suggested definitions focused on HIE (Table 6), which was seen as a potentially avoidable condition under ideal care conditions. Related or other frequently suggested definitions referred to causes of avoidable brain injury such as absent, poor, late or suboptimal clinical management during antenatal, intrapartum or perinatal care (Table 6). Participants noted that questions relating to avoidability should consider whether the outcome would have been different in a different healthcare unit, with different healthcare professionals, or using different clinical practices, while remaining cognisant of the challenges in determining avoidability.

**Table 6:** Participants’ recommendations when asked to characterise avoidable perinatal brain injury.

Type of definition	Examples
Specific definitions	Hypoxic ischaemic brain injury (*n* = 8) Lack of oxygen supplied to the baby (*n* = 3) Asphyxia (*n* = 2) Bleed/haemorrhagic event (*n* = 1)
Attribution-based definitions	Caused by poor or suboptimal perinatal, antenatal, or intrapartum management or (lack of, late, or suboptimal) care (*n* = 12)
General, broad and/or inclusive definitions	Insult or injury to the neonatal brain (*n* = 5) Certain types of obvious clinical damage relating to either primary brain but also multi-organ failure relating to ischaemic injury (*n* = 2) Injury that can cause short or long-term developmental delay or suboptimal outcomes (*n* = 2) Any intrapartum event associated with maternal or fetal conditions (*n* = 2)
Referential	Each Baby Counts report definition (*n* = 1), i.e. “severe brain injury diagnosed in the first 7 days of life, when the baby: (i) was diagnosed with grade III hypoxic ischaemic encephalopathy (HIE) OR (ii) was therapeutically cooled (active cooling only) OR (iii) had decreased central tone AND was comatose AND had seizures of any kind”

Participants identified the need for agreement on a set of data items relating clinical indicators and risk factors to generate an integrated data source on avoidable brain injury (Table 5 – theme 2). They suggested a large range of clinical indicators and risk factors to consider incorporating in an integrated source (Table 7). Some of these are already captured in current data sources as identified in our data dictionary review (see Tables 2 and 3), while others – particularly some intrapartum risk factors as recommended by (NICE)^54^ – are not. Participants strongly recommended that follow-up of babies with potential brain injury should be considered, since injury or developmental challenges may present some time after birth, even if there is no early indication of injury. They acknowledged that long-term follow-up may be challenging, and recommended exploring the use of existing community services (e.g. health visitors) and parents’ reports.

**Table 7:** Participants’ recommendations when asked to suggest data items relevant to monitoring incidence or risk factors for avoidable perinatal brain injury.

Clinical indicators of brain injury	Neonatal MRI (*n* = 12) Encephalopathy criteria (*n* = 3) Sarnat score (*n* = 1) Apgar and/or cord pH (*n* = 9), especially Apgar at 5 minutes (*n* = 1) Presence of convulsions (*n* = 11) Seizures (*n* = 3) EEG (*n* = 1) Difficulty in ventilating (*n* = 1) Evidence of Persistent Pulmonary Hypertension of the Newborn (*n* = 1)
Interventions indicative of efforts to avoid or reduce the impact of brain injury	Therapeutic cooling (*n* = 23) Therapeutic hyperthermia (*n* = 1) Resuscitation (*n* = 4) Respiratory effort (*n* = 1) Cord clamping and time of cord clamping (*n* = 1) Fetal scalp blood testing (*n* = 1) Support for breathing/circulation and drugs used in neonatal intensive care management (*n* = 1)
Postnatal/neonatal conditions or observations	Blood gas (*n* = 19) Any package of data indicators for neurological abnormalities (*n* = 2), such as the Glasgow Coma scale (*n* = 1) CFAM (*n* = 2) Decreased central tone, fitting, or altered tone (*n* = 3) Lactate or creatinine at first infant’s blood gas (*n* = 3) Baby’s behaviour (*n* = 2) Hypertonia, floppiness, and abnormal reflexes (*n* = 2) Primary sepsis (*n* = 2) Glucose levels (*n* = 1) Jaundice (*n* = 1) Hypoglycaemia (*n* = 1) Baby’s colour (*n* = 1) Kernicterus (*n* = 1) Comatose (*n* = 1) Blood sugars (*n* = 1) Thompson scoring (*n* = 1) Positive microbiology results (*n* = 1)
Service indicators	Transfer to a specialist unit (*n* = 14) Duration of stay in NICU (*n* = 11)
Maternal demographic and/or antenatal history	Woman’s health and risk factors such as demographics, morbidities, history, concurrent maternal medical conditions (*n* = 9) Antenatal history (*n* = 3) Women’s ethnicity and socioeconomic status, including social deprivation and BAME status (*n* = 1) Antenatal bleeding (*n* = 1) Ultrasound (*n* = 1) Multiple births, breech births (*n* = 1) Gestation (*n* = 2) Fetal growth restriction at the onset of labour (*n* = 6) Size, weight, ratio, and abdominal circumference (*n* = 1) Growth (*n* = 1) Brain sparing, end doppler, shrunken liver (*n* = 1)
Intrapartum risk factors	Duration of the first stage (*n* = 5) Duration of the second stage (*n* = 5) CTG classifications (*n* = 5) Meconium staining of the amniotic fluid (*n* = 5) Bleeding during labour (*n* = 3) Pyrexia (*n* = 3) Raised maternal temperature (*n* = 1) Duration of any cytokinin augmentation (*n* = 1) Fetal movements (*n* = 2) Presence of sentinel event (*n* = 1) Turtling (shoulder dystocia) (*n* = 1) Whether maternal heartbeat was considered normal or abnormal (*n* = 1) Whether abruption followed mother’s complaint of pain (*n* = 2)
Other	Mode of delivery (*n* = 2) Optiv records (e.g., whether caesarean birth, etc.) (*n* = 2) Intermittent auscultation (*n* = 1) Whether there was renal/liver injury and other secondary injuries (*n* = 1)

*BAME* Black, Asian, and Minority Ethnic, *CFAM* Cerebral Function Analysing Monitor, *CTG* cardiotocography, *EEG* electroencephalogram, *MRI* magnetic resonance imaging, *NICU* neonatal intensive care unit.

To enable and optimise an integrated data source, participants recommended addressing the duplication, inconsistency and subjectivity involved in capturing many data items relevant to brain injury (e.g. Apgar scores, therapeutic hypothermia criteria, fetal growth restriction, intrapartum risk factors) (Table 5 – theme 3). This would require a programme of work needed to operationalise and standardise operational definitions that are currently not harmonised across data sources and associated electronic patient record (EPR) platforms (Table 5 – theme 4). Further work would be needed to improve consistent, reliable and feasible data collection (Table 5 – theme 5), ranging from making the data collection system more user-friendly through to real-time capture of data rather than retrospective data inputting (Supplement 3), and training professionals involved in data capture (Table 5 – theme 6). Finally, participants identified the need for co-designing systems with healthcare professionals and families to improve use of data, including generating feedback loops that are meaningful to healthcare professionals and families based on sensible comparative methods (Table 5 – theme 7).

## Discussion

This review of UK perinatal data source dictionaries and multi-professional consultation identified challenges and opportunities in capturing and using data relating to avoidable perinatal brain injury associated to HIE. It shows many limitations of current data sources: none of them on its own captures the range of items relevant to injury, key intrapartum risk factors are not consistently captured, operational definitions of similar data items differ across data sources, and a mechanism for ongoing linkage of data across data sources is absent. Our consultation suggests that a first step towards improvement is to develop consensus on a standardised definition of avoidable perinatal brain injury and the range of clinical indicators and risk factors that should be captured in an integrated UK data source. A second step is to address current inconsistencies in capturing data across different data sources and healthcare units, with a focus on enabling systematic and ongoing linkage and integration of available data. Third, systems for better use of data should be co-designed with both healthcare professionals and families.

The challenges we identified are likely linked to the definitional morass that characterises the international and national field both in clinical guidelines and the wider literature,^10,39,52,54,61,66–68^ absence of UK standardisation in data collection and reporting,^21,30,69,70^ and fragmentation of data across different UK data sources and care pathways.^30,71^ A consensus-built integrated data source, based on a standardised definition for avoidable perinatal brain injury that could be informed by clinical criteria for HIE,^8–13,67^ would provide the foundation for identifying and defining risk factors potentially amenable to practice improvement. An integrated data source would also help in assessing the extent to which clinical indicators and risk factors for injury might be patterned by structural factors such as socio-economic disadvantage and ethnicity.^72,73^ Consensus work on a standardised definition and a set of data items for an integrated data course could be informed by ongoing definitional efforts in neonatology^74,75^ and neonatal encelopathy.^8,9^ It should include engaging with healthcare professionals and families to generate a data source that includes data items that are meaningful to professionals and families.^8,9,26,32,33,70,74–78^ The process and outcomes of this UK-focused work may inform other national and international perinatal audit processes and quality improvement efforts that also face challenges of limited standardisation or data fragmentation.^10,22,24,27,35,37,39,59,79^

Our consultation showed strong support for better and continuous linkage and integration of data captured across various UK data sources, electronic patient record platforms, and healthcare units. This resonates with previous findings of the NMPA^30^ and the British Association of Perinatal Medicine (BAPM).^46^ Linkage between data sources currently occurs on an ad-hoc basis, highlighting the challenges in consistently linking and integrating data from different sources.^30^ Although awareness is growing, further action by commissioners and policy-makers is needed to address the problem of electronic patient record platforms with different styles and formats, which risk worsening heterogeneity in data capture across maternity or neonatal units.^46^ Better aligned national data sources might also help improve international harmonisation efforts on perinatal data.^24,35,38^

Essential to the formation of an integrated data source is exploring possibilities for modern technology to facilitate safe data input and transfer across different platform providers. This is in line with national ambitions to create a more digitised and learning-focused NHS, guided by the principles of user need, privacy and security, interoperability and openness, and inclusion.^80^ How data should be extracted for local, national and potential international purposes requires further consultation, including families, maternity advocates, healthcare professionals, audit specialists, quality improvement practitioners, software developers, academics, and others who might be tasked with collecting, recording or reporting data.

Another key consideration for design of an integrated data source relates to when, how and by whom the data will be collected. Currently, complex transitional care arrangements differ between UK neonatal units,^71^ leading to variation in how neonatal data are captured in one of the current datasets and on whom. For example, in some cases babies are admitted under neonatal care and therefore have a neonatal dataset record with details captured by the NNRD. In other cases, data for the baby may only be entered into the mother’s local electronic patient record, with selected data items about the birth (e.g. Apgar score, resuscitation, seizures) subsequently captured by the MSDS. The need to address the “who” of data collection is also relevant to limited use of parent-reported symptoms of perinatal brain injury, which is an area in need of further exploration.

The development of an integrated UK data source built on consultation or co-design would support a learning health systems approach to improvement.^81^ For example, availability of a national integrated data source, coupled with a co-designed approach to data feedback and visualisation at local and national level, could provide the means for iterative, data-driven improvements through learning from routinely collected data.^81^ Despite the significant potential of a learning system approach,^81,82^ including for perinatal care,^83^ it is yet to be implemented in national perinatal settings.^82,83^ Investments would be needed, such as in resources and personnel required to monitor local, regional and national trends in brain injury incidence. These investments may not only help with better data capture, but also build professionals’ capability to reshape care, including reducing variations in quality and safety that contribute to avoidable brain injury.^3,5,6^

### Strengths and limitations

This paper offers insights into the potential for current UK perinatal data sources to support improvement in care relating to avoidable perinatal brain injury associated to HIE, including the generation of an integrated UK data source based on harmonised definitions. It also synthesises the views of diverse professionals regarding challenges and suggestions for developing such a data source. Its methods may be useful for other clinical areas in which national or international sources of routinely collected data could inform healthcare improvement activities, but this requires further evaluation.

The review is limited by its focus on national data sources (that is, not reviewing data captured in unit-level databases), and an inability to collect accurate information on the quality, completeness and sample size of data captured in the data sources. Although the multi-professional consultation included a varied sample with a multitude of views that resonate with recommendations by other expert groups,^30,46^ it is possible that wider consultation could further strengthen or expand the conclusions in this paper. Families and those affected by perinatal brain injury were not involved during this initial phase owing to its technical nature, but will be crucial to a next phase of work of a programme to develop a data source that addresses the priorities and views of families and professionals.

## Conclusion

This study has identified challenges in monitoring incidence and risk factors of avoidable perinatal brain injury in the UK, including problems in standardisation and integration of routinely collected data across different sources. These challenges limit the value and utility of current data sources as a basis for improvement efforts. A strategy including consultation, consensus-building, and co-design with healthcare professionals, families, and other specialists is needed to harmonise data definitions and ensure reliable and feasible ways to capture data. This strategy should aim to ensure that all perinatal events are reported using the same definitions and according to appropriate quality standards. Work to develop a high-quality integrated source should be accompanied by co-design to enable use of data to understand problems, generate solutions, and test solutions as part of improvement efforts.

## Supplementary information

Supplementary Material

## Data Availability

The data that support the findings of this evaluation are available on request from the corresponding author. Access to fully anonymised data may be granted to bona fide researchers under a data sharing agreement. The data are not publicly available due to privacy or ethical restrictions.
