# Correction: Improving UK data on avoidable perinatal brain injury: review of data dictionaries and consultation

**DOI:** 10.1038/s41390-025-03949-7

**Published:** 2025-02-24

**Authors:** Jan W. van der Scheer, Victoria Komolafe, Kirstin Webster, Stamatina Iliodromiti, Charles C. Roehr, Asma Khalil, Tim Draycott, Louise Dewick, George Dunn, Rachel Walsh, Philip Steer, Alessandra Giusti, Mark L. Cabling, Nick Fahy, ABC Contributor Group, Alissa E. Frémeaux, Amar M. Karia, Annette Anderson, Bertie Leigh, Chris Gale, Cora Doherty, Daniel Wolstenholme, James Walker, Julia Gudgeon, Laura Cowell, Marian Knight, Matthew C. Jolly, Muhammed Ally Hussein Wahedally, Tim J. van Hasselt, Tina Harris, Mary Dixon-Woods

**Affiliations:** 1 THIS Institute (The Healthcare Improvement Studies Institute), University of Cambridge, Strangeways Research Laboratory, Cambridge, CB1 8RN UK; 2 Royal College of Midwives, London, SE1 1SZ UK; 3 Royal College of Obstetricians & Gynaecologists, 10-18 Union Street, London, SE1 1SZ UK; 4 Department of Population Sciences, University of Leicester, Leicester, LE1 7RH UK; 5 Women’s Health Research Unit, Wolfson Institute of Population Health, Queen Mary University London, London, UK; 6 Faculty of Health Sciences, University of Bristol, Bristol, UK; 7 National Perinatal Epidemiology Unit, Clinical Trials Unit, Oxford Population Health, Medical Sciences Division, University of Oxford, Oxford, UK; 8 North Bristol NHS Trust, Bristol, BS10 5NB UK; 9 NHS Resolution, E14 4PU London, UK; 10 RAND Europe Westbrook Centre Milton Road, Cambridge, CB4 1YG UK; 11 Hempsons, London, UK; 12 Imperial College London, London, UK; 13 Health Services Safety Investigations Body, Dorset, UK; 14 NHS England, London, UK; 15 University of Oxford, Oxford, UK; 16 University of Leicester, Leicester, UK; 17 De Montfort University, Leicester, UK

Correction to: *Pediatric Research* 10.1038/s41390-025-03842-3, published online 30 January 2025

In this article, the author’s name Stamatina Iliodromiti was incorrectly written as Stamatina Iliodriomiti. The original article has been corrected.

