# Supplementary material for: Improving UK data on avoidable perinatal brain injury: review of data dictionaries and consultation
Source: Pediatr Res. 2025 Jan 30;98(3):853–63. doi: 10.1038/s41390-025-03842-3 (PMC12507660; doi:10.1038/s41390-025-03842-3)
Supplement: Supplementary file 1 — Supplementary Material [file 41390_2025_3842_MOESM1_ESM.pdf]

Supplement 1

Interview prompt guide

The Department of Health and Social care has asked us to look at the data we need to collect so as assess the baby's condition during labour and the neonatal period, and thereby inform our efforts to prevent avoidable brain injury.

1. What do you understand by the term 'avoidable brain injury'?

2. What data items do you think we would need to collect in the neonatal period in order to decide whether a baby had potentially avoidable brain injury?
   (The following examples to be used as prompts: Convulsions post-delivery, Cooling, Blood gases at birth, Duration of stay in NICU, Transfer to specialist unit)

3. Which of the following data items do you think it would be necessary for us to collect to characterise 'potentially avoidable brain injury':
   a. Apgar score at 5 minutes after birth
   b. Apgar score at 10 minutes after birth
   c. Cord blood artery and vein pH and base deficit
   d. Clinical seizures
   e. Hypotonia (floppiness)
   f. Abnormal reflexes
   g. Absent or weak suck
   h. Electroencephalogram (EEG) abnormal

4. Can you think of any other data items that might be necessary?

5. Do you think we should follow up all babies for a minimum period, such as six weeks, and how could that be done? What would need to be recorded about their status?

6. Do you think it would be useful if the above data items were retrospectively entered into maternity databases, and how feasible do you think that would be?

7. Do you think it would be useful if the above data were routinely reported to the DHSC so that they could feed back to each maternity unit on a regular basis the incidence of such indicators in their unit relative to a national average?

8. At the moment, most nationally collected datasets contain very few indicators of fetal wellbeing during labour. Of the following (all of which are currently missing from national obstetric datasets), which do you think should be collected:

   a. Suspected fetal growth restriction at the onset of labour
   b. Duration of the first stage of labour
   c. Duration of the second stage of labour
   d. Meconium staining of the amniotic fluid

e. Bleeding during labour
f. Pyrexia (maximum maternal temperature in labour)
g. Fetal heart rate normal/abnormal

9. In relation to item g above, how feasible and appropriate would it be to collect:

a. Highest baseline rate
b. Abnormal baseline variability
c. Repetitive decelerations (regardless of type)
d. Acute prolonged bradycardia#

10. What would need to happen to record intrapartum brain injury reliably on maternity databases (e.g. from the perspective of computer architecture and system suppliers, staff time to collect and report the data, cultural changes etc etc)?

11. What organisational change would be required for these data to be collected consistently and reliably across all maternity/neonatal services? If you were in charge, would you recommend additional funding/resources/staffing? If so, please outline them.

12. What do you think would be the best way to feedback information about the data items described above to managers and clinicians, once they have been collected? Would it be best as tables, graphs, or illustrative cartoons? Or some other method? (Please suggest)

Supplement 2

Interview data analysis

The interviews (lasting 22 to 90 minutes) were transcribed verbatim, and anonymised for identifying information about individuals or organisations.

Thematic analysis was guided by Braun and Clarke's proposed approach [see 1], and consisted of six steps:

(1) A healthcare service analyst (MC) used the questions of the interview topic guide as an initial thematic framework to categorise relevant quotes from all interviews.
(2) The analyst generated a longlist list of potential themes cross-cutting the interview questions, including illustrative quotes associated to each theme.
(3) The longlist informed discussion among the analyst, senior analyst (NF), lead author (JvdS) and senior author (MDW) to generate a final thematic framework.
(4) The analyst (MC) used the framework to categorise relevant quotes from all interviews, and generated draft summaries for each theme.
(5) The lead author and senior author verified and refined the summaries, working iteratively with the analyst to ensure that summaries accurately reflected the data.
(6) The authorial team iteratively synthesised the summaries, informed by their professional expertise and relevant literature.

1. Braun V, Clarke V. Is thematic analysis used well in health psychology? A critical review of published research, with recommendations for quality practice and reporting. Health Psychol Rev 2023:1-24. doi: 10.1080/17437199.2022.2161594 [published Online First: 2023/01/20]

Supplement 3

Examples of participants' recommendations on how to make data collection more feasible for healthcare professionals during provision of routine care.*

Recommendation to make data collection more feasible	Number of experts
Adopt data flow innovations that have been successful in other disciplines or that already successfully exist within the National Health Service (NHS)	5
Encourage engagement with data through integrated but simple dashboards that prompt questions more actively for professionals	5
Involve data managers (e.g. digital midwives) in the design of data collection systems and interfaces so that they are user friendly	4
Stray away from manual or handwritten notetaking due to difficulties with digitalisation (if used, ensure option to skip data item)	4
Rely more on electronic systems to do the interpretation of data	2
Allow only pre-defined data, such as tick boxes instead of filling out data through free text	2
Prioritise collecting data items that are easiest to record and readily available during routine care	1

*See Table 4 – "Organisational change to improve consistent, reliable and feasible data collection"
